# The effect of emphysema on readmission and survival among smokers with heart failure

**DOI:** 10.1371/journal.pone.0201376

**Published:** 2018-07-30

**Authors:** Puja Kohli, Pedro V. Staziaki, Sumbal A. Janjua, Daniel A. Addison, Travis R. Hallett, Orla Hennessy, Richard A. P. Takx, Michael T. Lu, Florian J. Fintelmann, Marc Semigran, Robert S. Harris, Bartolome R. Celli, Udo Hoffmann, Tomas G. Neilan

**Affiliations:** 1 Pulmonary and Critical Care Unit, Department of Medicine, Massachusetts General Hospital, Boston, Massachusetts, United States of America; 2 Cardiac MR PET CT Program, Department of Radiology, Massachusetts General Hospital, Boston, Massachusetts, United States of America; 3 Division of Thoracic Imaging and Intervention, Department of Radiology, Massachusetts General Hospital, Boston, Massachusetts, United States of America; 4 Division of Cardiology, Department of Medicine, Massachusetts General Hospital, Boston, Massachusetts, United States of America; 5 Division of Pulmonary and Critical Care Medicine, Department of Medicine, Brigham & Women’s Hospital, Boston, Massachusetts, United States of America; National and Kapodistrian University of Athens, SWITZERLAND

## Abstract

Heart Failure (HF) and chronic obstructive pulmonary disease (COPD) are morbid diseases that often coexist. In patients with coexisting disease, COPD is an independent risk factor for readmission and mortality. However, spirometry is often inaccurate in those with active heart failure. Therefore, we investigated the association between the presence of emphysema on computed tomography (CT) and readmission rates in smokers admitted with heart failure (HF). The cohort included a consecutive group of smokers discharged with HF from a tertiary center between January 1, 2014 and April 1, 2014 who also had a CT of the chest for dyspnea. The primary endpoint was any readmission for HF before April 1, 2016; secondary endpoints were 30-day readmission for HF, length of stay and all-cause mortality. Over the study period, there were 225 inpatient smokers with HF who had a concurrent chest CT (155 [69%] males, age 69±11 years, ejection fraction [EF] 46±18%, 107 [48%] LVEF of < 50%). Emphysema on CT was present in 103 (46%) and these were older, had a lower BMI, more pack-years, less diabetes and an increased afterload. During a follow-up of 2.1 years, there were 110 (49%) HF readmissions and 55 (24%) deaths. When separated by emphysema on CT, any readmission, 30-day readmission, length of stay and mortality were higher among HF patients with emphysema. In multivariable regression, emphysema by CT was associated with a two-fold higher (adjusted HR 2.11, 95% CI 1.41–3.15, p < 0.001) risk of readmission and a trend toward increased mortality (adjusted HR 1.70 95% CI 0.86–3.34, p = 0.12). In conclusion, emphysema by CT is a frequent finding in smokers hospitalized with HF and is associated with adverse outcomes in HF. This under recognized group of patients with both emphysema and heart failure may benefit from improved recognition and characterization of their co-morbid disease processes and optimization of therapies for their lung disease.

## Introduction

Heart failure and chronic obstructive pulmonary disease (COPD) are both global epidemics that incur significant morbidity and mortality [1]. These systemic disorders often coexist with overlapping clinical symptoms and pathophysiologic processes [2]. Few have studied the prognostic implications of comorbid illness with HF and COPD, but these studies have shown that the presence of COPD is an independent predictor of death and HF hospitalization [2]. However, given the overlapping clinical signs and symptoms in this population and inaccurate spirometry measurements in patients with active heart failure [3,4], COPD is likely underdiagnosed.

While the care of patients with HF has improved dramatically [5], there is room for continued improvement [6]. Specifically, readmission after a HF hospitalization is still high [7]. For example, data have shown that within 30 to 60 days of discharge 30% of patients with HF will be re-hospitalized [8]. Reducing heart failure hospital readmissions is paramount for improved patient care and health care costs.

Emphysema is pathological destruction of alveoli leading to lung hyperinflation on chest CT, and is seen in 50% of patients with chronic obstructive pulmonary disease (COPD) [9] and in an estimated 44% of asymptomatic smokers [10]. Emphysema can be diagnosed using either pulmonary function testing (PFTs) or computed tomography (CT). However, data suggest that emphysema by CT may be a more sensitive measure of lung disease [11], especially in HF, may be a better predictor of adverse outcomes [12,13] and is associated with a lower cardiac output [14–16].

Despite the plausibility [17–23], there are no data that either test the effect of emphysema by CT on adverse outcomes among patients with complex HF or provide potential mechanistic insight in this population. In this study we aimed to add to this limited data and hypothesized that emphysema by CT is associated with an increase in hospital readmission and mortality among patients with complex HF.

## Methods

This was a historical-cohort single-center study. This investigation conforms with the principles outlined in the *Declaration of Helsinki* and the research protocol was approved by the Partners Human Research Committee (PHRC), the Institutional Review Board (IRB) of Partners Healthcare, which waived the need for informed consent. The cohort was derived from the Partners HealthCare System Research Patient Data Registry (RPDR), a clinical care database of all inpatient and outpatient encounters from Massachusetts General Hospital. We queried RDPR for all patients with a primary discharge diagnosis of HF from January 1^st^ to April 1^st^ 2014. The cohort was then restricted to active or prior smokers and those who had a contemporary CT scan of the chest (within 2 years of HF discharge) performed for dyspnea. A discharge diagnosis of HF was adjudicated by investigators blinded to all other variables using the ACC/AHA definition of HF [24]. In total, three independent blinded groups performed each of the key components: clinical data collection, emphysema by CT, and event adjudication. The presence of emphysema was assessed using a CT scan of the chest by a board-certified pulmonologist blinded to all other data using validated criteria from the National Emphysema Treatment Trial [25]. In brief, using inspiratory CT scans, each lung was divided into three apical-to-basal zones, and each zone was evaluated visually. Emphysema was diagnosed by CT if at least one of the zones showed emphysematous lucencies in greater than 5% of the lung zone (grade 1).

The primary endpoint was the first readmission for HF after the index hospitalization for HF. The occurrence of a HF admission was defined based on ACC/AHA guidelines [24]. The principal secondary end-point was all-cause mortality determined by electronic medical record. Two additional secondary end-points were pre-specified: 30-day readmission for HF and length of stay for the first readmission with HF.

Covariates were recorded at the time of the index hospitalization. In brief, cardiovascular risk factors collected included smoking history, hypertension, dyslipidemia, diabetes mellitus, presence of coronary artery disease, sleep apnea, body mass index (BMI), stroke or cerebrovascular accident and atrial fibrillation. Sleep apnea was defined based on sleep study criteria recommended by the American Academy of Sleep Medicine [26]. Stroke or cerebrovascular accident were defined as documented history of ischemic stroke, silent central nervous system (CNS) infarction, intracerebral hemorrhage, silent intracerebral hemorrhage, subarachnoid hemorrhage, central venous thrombosis, evidence of CNS infarction on imaging or pathology, or any episode of acute neurological dysfunction presumed to be caused by ischemia or hemorrhage, persisting ≥24 hours or until death [27]. Cardiovascular medications and ECG and echocardiogram data were also recorded. ECG parameters included presence of sinus rhythm, PR interval (msec), QRS width (msec) and the QTc (msec) interval. Echocardiographic parameters included the left atrial dimension (LA, mm), pulmonary artery systolic pressure (PASP, mmHg), posterior wall thickness in systole and diastole (PWT, mm), left ventricular ejection fraction (LVEF, %) and end-systolic wall stress as a measure of cardiac afterload. End-systolic wall stress was calculated based on American Society of Echocardiography recommendations [28]. Pulmonary specific factors included pulmonary function tests and lung medications.

Baseline characteristics, available for all participants, grouped by the presence or absence of emphysema, are presented as frequency (percentage) for categorical data and mean ± standard deviation or median (interquartile ranges [IQR]) for continuous data unless otherwise stated. Continuous data were compared with the use of unpaired Student t tests or Wilcoxon rank-sum tests when appropriate. Categorical data were compared using the chi-square or the Fisher exact test. Univariable and multivariable Cox regression analysis was performed. The hazard ratio for the prediction of events was calculated for first readmission, 30-day readmission, and for mortality rates using Cox proportional hazard models. In the multivariate analysis, we used two Cox regression models. In the first model, we included the predictors of readmission that had a p value < 0.1 on the univariate analysis. The second multivariable model was based on known predictors of HF hospitalization and emphysema and included age [29–32], gender [33], diabetes [32,34], hypertension [35], CAD [35], obstructive sleep apnea, chronic kidney disease [36–39], ejection fraction and sodium [40]. The proportional-hazards assumption was met in all models, and all models fit the data well. Event curves were determined according to the Kaplan-Meier method, and comparisons of cumulative event rates were performed by the log-rank test. Intervals were defined from date of index discharge. The end of the observation period included the earlier of two possible termination points: death or last encounter prior to April 1^st^ 2016. All analyses were performed using R (Version 3.2.2, The R Foundation for Statistical Computing Platform) and a two-sided p value of < 0.05 was considered statistically significant.

## Results

**Fig 1:**
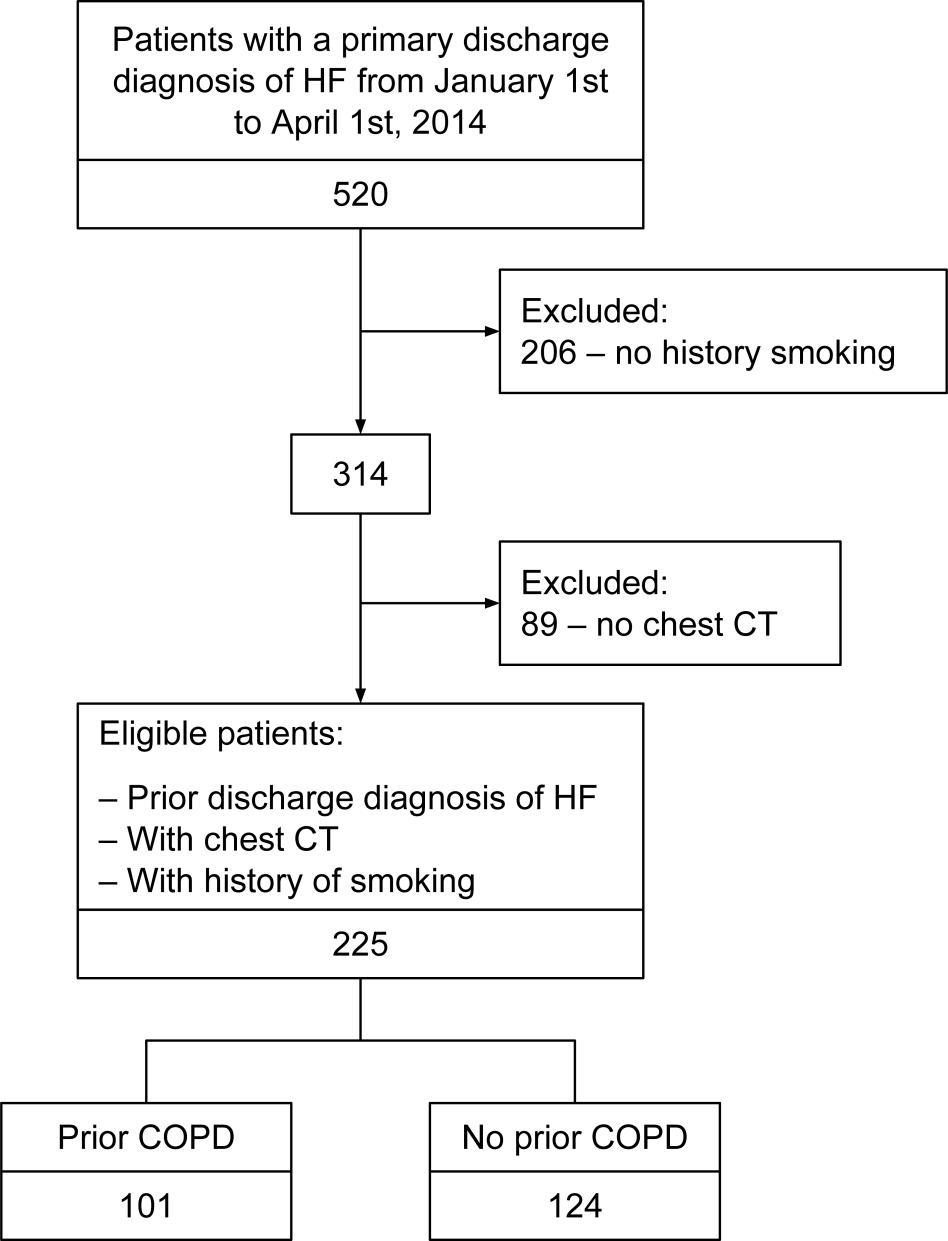
Flow diagram. Flow diagram describing the inclusion and exclusion criteria for the study population.

The RPDR query yielded 520 patients with a primary adjudicated discharge diagnosis of HF from January 1^st^ to April 1^st^ 2014 (Fig 1). Overall, there were 314 current or past smokers among the 520 subjects discharged from this facility over a 3-month period with HF. Subsequently, patients without a contemporary CT scan (91 subjects) were excluded leaving a final study cohort of 225 smokers with adjudicated HF discharged from an academic center over a 3-month period with a recent chest CT. Complete details of the cohort are presented in Table 1. In brief, the mean age was 69 ± 11 years, there were 155 (69%) males, 167 (74%) had hypertension, 111 (49%) had diabetes and 123 (55%) had CAD and the average number of pack-years was 39. In total, 101 (45%) had a clinician diagnosis of COPD and the average predicted FEV1 among that population was 63% ± 20. The average LVEF was 47% ± 18 and 107 (48%) had an LVEF of < 50%. The cohort was separated by the presence or absence of emphysema on CT (Table 1). Overall, 103 (46%) patients had emphysema on CT (Table 1). Patients with emphysema on CT were older, had a lower BMI, a higher number of pack-years, more likely to have clinician diagnosed COPD, less likely to have diabetes and had a markedly increased afterload (end-systolic wall stress). Other co-morbidities, use of standard HF medications and LVEF were similar between groups. There were 50 patients (48%) in the emphysema group with an LVEF of < 50%. Among those with PFTs (n = 80), there was a significant difference in the forced expiratory volume in 1 second (FEV1) to forced vital capacity (FVC) ratio between those with and without emphysema by CT (FEV1/FVC 63 vs 74, p = 0.0004). However there was no difference between the FEV1 or the predicted residual volume. (Table 2) Among the 103 patients with emphysema on CT there were 40 patients (39%) who did not have a clinical diagnosis of COPD, whereas out of 104 patients with clinical diagnosis of COPD there were 38 patients (36.5%) who did not have emphysema on CT.

**Table 1:** Patient characteristics by emphysema status.

		All patients	Emphysema, no	Emphysema, yes	
Variables		n = 225	n = 122	n = 103	p Value
**Age**		69 ± 11	68 ± 11	71 ± 10	0.02
**Male**		155 (69)	88 (72)	67 (65)	0.25
**BMI**		30 ± 8.2	32 ± 8.9	28 ± 6.9	0.01
**Pack-years**		39 ± 28	32 ± 23	48 ± 32	< 0.001
**Prior medical conditions**					
	**Hypertension**	167 (74)	95 (78)	72 (70)	0.17
	**Diabetes mellitus**	111 (49)	71 (58)	40 (39)	0.004
	**Dyslipidemia**	121 (54)	66 (54)	55 (53)	0.92
	**Chronic kidney disease**	61 (27)	39 (32)	22 (21)	0.07
	**Obstructive sleep apnea**	36 (16)	19 (16)	17 (16)	0.84
	**Valvular disease**	50 (22)	22 (18)	28 (27)	0.10
	**CAD**	123 (55)	66 (54)	57 (55)	0.85
	**PAD**	26 (11)	12 (9.8)	14 (14)	0.38
	**Stroke**	29 (13)	19 (15)	10 (9.7)	0.19
	**Atrial fibrillation**	105 (47)	61 (50)	44 (43)	0.27
**Chronic lung diseases**					
	**COPD**	104 (45)	38 (31)	63 (61)	< 0.001
	**Asthma**	15 (6.6)	10 (8.1)	5 (4.8)	0.32
**Pulmonary medications at discharge**					
	**Inhaled steroids**	29 (13)	13 (11)	16 (15)	0.28
	**Inhaled beta-agonists**	75 (33)	35 (29)	40 (39)	0.11
	**Inhaled LA antimuscarinic**	54 (24)	18 (15)	36 (35)	< 0.001
	**Oral corticosteroids**	19 (8.4)	7 (6.7)	12 (12)	0.11
**Other medications at discharge**					
	**Aspirin**	177 (79)	95 (78)	82 (80)	0.75
	**ACEI/ARB**	178 (79)	91 (74)	87 (84)	0.07
	**Beta-blocker**	203 (90)	113 (93)	90 (87)	0.19
	**Calcium channel blocker**	42 (19)	27 (22)	15 (15)	0.15
	**Statin**	172 (76)	93 (76)	79 (77)	0.93
	**Aldosterone antagonist**	50 (22)	32 (26)	18 (17)	0.11
	**Diuretics**	207 (92)	112 (92)	95 (92)	0.91
	**Nitrates**	41 (18)	24 (20)	17 (16)	0.54
	**Digoxin**	28 (12)	11 (9)	17 (16)	0.09
	**Coumadin**	88 (39)	47 (38)	41 (40)	0.84
**Heart rate (bpm)**		81 ± 17	82 ± 18	81 ± 17	0.83
**Systolic blood pressure (mm Hg)**		132 ± 27	132 ± 27	131 ± 28	0.75
**Diastolic blood pressure (mm Hg)**		72 ± 13	72 ± 14	71 ± 13	0.68
**Laboratory values**					
	**Sodium**	137 ± 4.1	137 ± 4.3	137 ± 3.8	0.57
	**Creatinine**	1.3 (1.0–1.8)	1.3 (1.0–2.0)	1.3 (1.0–1.8)	0.63
	**Blood urea nitrogen**	31 (19–45)	32 (19–47)	28 (17–41)	0.11
**ECG**					
	**Non-sinus rhythm**	81 (36)	45 (39)	36 (38)	0.94
	**P-R interval (msec)**	174 ± 34	177 ± 35	170 ± 33	0.23
	**QRS duration (msec)**	119 ± 35	120 ± 35	117 ± 35	0.41
	**QTc (msec)**	474 ± 50	476 ± 46	472 ± 54	0.52
**Cardiac ultrasound**					
	**LA dimension (mm)**	45 ± 7.0	46 ± 6.6	45 ± 7.5	0.19
	**LVIDd (mm)**	52 ± 9	52 ± 9	52 ± 9	0.70
	**LVIDs (mm)**	40 ± 11	40 ± 11	40 ± 11	0.96
	**PWT (mm)**	11 ± 2.2	11 ± 2.1	11 ± 2.4	0.29
	**SWT or IVS (mm)**	12 ± 2.6	12 ± 2.6	12 ± 2.6	0.86
	**LVEF (%)**	47 ± 18	47 ± 18	46 ± 18	0.74
	**PASP (mm Hg)**	46 ± 14	46 ± 14	46 ± 14	0.89
	**ESWS**	89 ± 41	78 ± 34	102 ± 45	< 0.001

BMI = body mass index, CAD = coronary artery disease, PAD = peripheral arterial disease, COPD = chronic obstructive pulmonary disease, ILD = interstitial lung disease, FEV1 = predicted forced expiratory volume in 1 second, FVC = forced vital capacity, RV = predicted residual volume, DLCO = diffusing capacity of the lung for carbon monoxide, LA = long-acting, ACEI = angiotensin-converting enzyme inhibitor, ARB = angiotensin II receptor blocker, NOAC = novel oral anticoagulants, ECG = electrocardiogram, LA = left atrium, PWT = posterior wall thickness, SWT = septal wall thickness, LVEF = left ventricular ejection fraction, PASP = pulmonary artery systolic pressure, ESWS = end-systolic wall stress

**Table 2:** Pulmonary function testing.

Variables	All Tested Patients n = 80	Emphysema, no n = 35	Emphysema, yes n = 45	p-value
**FEV1 (%)**	63 ± 20	64 ± 18	62 ± 21	0.58
**FVC (%)**	57 ± 33	54 ± 31	59 ± 35	0.55
**FEV1/FVC**	68 ± 12	74 ± 9	63 ± 12	0.0004
**RV (%)**	109 ± 40	92 ± 37	122 ± 41	0.13
**DLCO (%)**	52 ± 21	59 ± 23	45 ± 18	0.01

In Table 3 we compared covariates between those readmitted with HF and those not readmitted with HF within the duration of the study. As compared to those not re-admitted with HF, those readmitted had decreased usage of inhaled anti-muscarinics. All other prior medical conditions and medications were similar between the two groups.

**Table 3:** Patient characteristics by HF readmission status.

		All patients	No readmission	Readmission	
Variables		n = 225	n = 110	n = 115	p Value
**Age**		69 ± 11	69 ± 11	69 ± 11	0.73
**Male**		155 (69)	74 (64)	81 (74)	0.13
**BMI**		30 ± 8.2	30 ± 8.6	31 ± 7.8	0.44
**Pack-years**		39 ± 28	37 ± 27	41 ± 30	0.27
**Prior medical conditions**					
	**Hypertension**	167 (74)	84 (73)	83 (75)	0.68
	**Diabetes mellitus**	111 (49)	61 (53)	50 (45)	0.25
	**Dyslipidemia**	121 (54)	59 (51)	62 (56)	0.45
	**Chronic kidney disease**	61 (27)	32 (29)	29 (26)	0.80
	**Obstructive sleep apnea**	36 (16)	16 (14)	20 (18)	0.38
	**Valvular disease**	50 (22)	24 (21)	26 (24)	0.62
	**CAD**	123 (55)	59 (51)	64 (58)	0.30
	**PAD**	26 (11)	12 (10)	14 (13)	0.59
	**Stroke**	29 (13)	17 (15)	12 (11)	0.39
	**Atrial fibrillation**	105 (47)	58 (50)	47 (43)	0.25
**Chronic lung diseases**					
	**COPD**	104 (45)	46 (40)	55 (50)	0.13
	**Asthma**	15 (6.6)	10 (8.7)	5 (4.5)	0.21
**Pulmonary function tests**					
	**FEV1 (%)**	63 ± 20	64 ± 20	61 ± 19	0.52
	**FVC (%)**	57 ± 33	67 ± 28	49 ± 34	0.02
	**RV (%)**	109 ± 40	109 ± 59	109 ± 29	0.98
	**DLCO (%)**	52 ± 21	47 ± 20	55 ± 22	0.13
**Pulmonary medications at discharge**					
	**Inhaled steroids**	29 (13)	18 (16)	11 (10)	0.21
	**Inhaled beta-agonists**	75 (33)	43 (37)	32 (29)	0.19
	**Inhaled LA antimuscarinic**	54 (24)	34 (30)	20 (18)	< 0.05
	**Oral corticosteroids**	19 (8.4)	12 (10)	7 (6.4)	0.27
**Other medications at discharge**					
	**Aspirin**	177 (79)	91 (79)	86 (78)	0.86
	**ACEI/ARB**	178 (79)	92 (80)	86 (78)	0.74
	**Beta-blocker**	203 (90)	106 (92)	97 (88)	0.31
	**Calcium channel blocker**	42 (19)	18 (16)	24 (22)	0.23
	**Statin**	172 (76)	88 (76)	84 (76)	0.98
	**Aldosterone antagonist**	50 (22)	29 (21)	21 (19)	0.27
	**Diuretics**	207 (92)	104 (90)	103 (94)	0.38
	**Nitrates**	41 (18)	19 (16)	22 (20)	0.50
	**Digoxin**	28 (12)	13 (11)	15 (14)	0.60
	**Coumadin**	88 (39)	45 (39)	43 (39)	0.99
**Heart rate (bpm)**		81 ± 17	81 ± 17	82 ± 18	0.64
**Systolic blood pressure (mm Hg)**		132 ± 27	129 ± 26	134 ± 28	0.23
**Diastolic blood pressure (mm Hg)**		72 ± 13	71 ± 14	73 ± 13	0.21
**Laboratory values**					
	**Sodium**	137 ± 4.1	137 ± 4.0	136 ± 4.0	0.92
	**Creatinine**	1.3 (1.0–1.8)	1.3 (1.0–1.8)	1.2 (0.9–1.8)	0.22
	**Blood urea nitrogen**	31 (19–45)	29 (19–41)	31 (18–48)	0.50
**ECG**					
	**Non-sinus rhythm**	81 (36)	37 (34)	44 (43)	0.19
	**P-R interval (msec)**	174 ± 34	176 ± 37	172 ± 31	0.46
	**QRS duration (msec)**	119 ± 35	117 ± 34	120 ± 36	0.53
	**QTc (msec)**	474 ± 50	472 ± 46	477 ± 54	0.49
**Cardiac ultrasound**					
	**LA dimension (mm)**	45 ± 7.0	45 ± 7.6	46 ± 6.4	0.12
	**LVIDd (mm)**	52 ± 9	51 ± 9	53 ± 9	0.20
	**LVIDs (mm)**	40 ± 11	40 ± 11	40 ± 11	0.91
	**PWT (mm)**	11 ± 2.2	11 ± 2.0	11 ± 2.4	0.25
	**SWT or IVS (mm)**	12 ± 2.6	12 ± 2.3	12 ± 2.9	0.80
	**LVEF (%)**	46 ± 18	47 ± 17	47 ± 19	0.99
	**PASP (mm Hg)**	46 ± 14	45 ± 15	46 ± 13	0.69
	**ESWS**	89 ± 41	87 ± 41	90 ± 41	0.56

BMI = body mass index, CAD = coronary artery disease, PAD = peripheral arterial disease, COPD = chronic obstructive pulmonary disease, ILD = interstitial lung disease, LA = long-acting, ACEI = angiotensin-converting enzyme inhibitor, ARB = angiotensin II receptor blocker, NOAC = novel oral anticoagulants, ECG = electrocardiogram, LA = left atrium, PWT = posterior wall thickness, SWT = septal wall thickness, LVEF = left ventricular ejection fraction, PASP = pulmonary artery systolic pressure, ESWS = end-systolic wall stress

Follow-up was available for all subjects and the median follow-up period was 1.3 (IQR 1.1–1.4) year. During follow-up, 55 patients died (24% of cohort). There were 35 (64%) deaths among the 103 patients with emphysema as compared to 20 (36%) deaths among the 122 patients without (p = 0.002). The median time to death was 448 days in the full cohort, 440 days in patients with emphysema on CT vs. 453 days in the patients without emphysema on CT (Table 4). Patients with emphysema on CT had a higher likelihood of death regardless of whether they had co-existant heart failure with preserved ejection fraction (OR 3.4, p = 0.01) or reduced ejection fraction (OR = 2.75, p = 0.04). (Table 5)

**Table 4:** Outcomes.

		All patients	Emphysema, no	Emphysema, yes	
Variables		n = 225	n = 122	n = 103	p value
**Follow-up time**		466 (414–529)	469 (418–525)	464 (376–510)	0.64
**Death**		55 (24)	20 (36)	35 (64)	**< 0.001**
	**Time to death**	448 (268–507)	453 (343–515)	440 (249–500)	0.38
**Any readmission**		110 (49)	46 (42)	64 (58)	**< 0.001**
	**Time to readmission**	224 (59–451)	327 (69–477)	162 (31–323)	**0.003**
**30-day readmission**		36 (16)	11 (9)	25 (24)	**0.002**

**Table 5:** Outcomes by cardiac function.

**HF with Preserved EF > 50%**				
	**All patients**	**Emphysema, no**	**Emphysema, yes**	
**Variables**	**n = 115**	**n = 64**	**n = 51**	**p value**
**Death**	27 (23)	9 (7)	18 (35)	**0.01**
**Readmission**	60 (52)	23 (35)	37 (73)	**< 0.001**
**30-day readmission**	25 (22)	8 (12)	17 (33)	**0.01**
**HF with Reduced EF < 50%**				
	**All patients**	**Emphysema, no**	**Emphysema, yes**	
**Variables**	**n = 110**	**n = 57**	**n = 53**	**p value**
**Death**	27 (25)	9 (16)	28 (53)	**0.04**
**Readmission**	53 (48)	24 (42)	29 (55)	**0.25**
**30-day readmission**	14 (13)	5 (9)	9 (17)	**0.25**

HF = heart failure, EF = ejection fraction

In this cohort, there were 110 subjects (49%) readmitted with HF. The median time to first readmission with HF after the index hospitalization was 224 days. Overall, 64 of 103 patients (58%) with emphysema were readmitted with HF as compared to 46 of 122 patients (42%; p < 0.001). The median time to readmission for decompensated HF was 162 days in the patients with emphysema vs. 327 days in the patients without emphysema. (Table 4)

Overall, 36 (16%) patients were readmitted in 30 days or less. There were 25 (24%) 30-day readmissions among the 103 patients with emphysema as compared to 11 (9%) among the 122 patients without (p = 0.002). The median length of stay once readmitted with HF was longer among patients with emphysema than those without (7 days, IQR 5–8 vs. 5 days, IQR 3–8, p = 0.004). (Table 4)

In the univariable model, only emphysema on CT was associated with readmissions (Table 6, HR 2.07, 95% CI 1.42–3.04, p < 0.001). In the multivariable model adjusting for factors known to be predictive of readmission and mortality (Model 2), the presence of emphysema on CT was independently associated with an increased risk of readmission (Table 6, HR 2.11, 95% CI 1.41–3.15, p < 0.001).

**Table 6:** Cox proportional hazards models for time to readmission.

	Univariate analysis		Multivariate analysis (Model 2)	
	HR (95% CI)	p Value	HR (95% CI)	p Value
**Emphysema**	2.07 (1.42–3.04)	< 0.001	2.11 (1.41–3.15)	< 0.001
**Age**	1.01 (0.99–1.02)	0.44	1.00 (0.98–1.02)	0.98
**Sex**	1.26 (0.82–1.92)	0.29	1.35 (0.86–2.11)	0.19
**Hypertension**	1.02 (0.66–1.58)	0.92	1.06 (0.68–1.67)	0.78
**Diabetes mellitus**	0.82 (0.57–1.20)	0.31	0.93 (0.63–1.39)	0.73
**Chronic kidney disease**	0.96 (0.63–1.48)	0.88	0.96 (0.62–1.49)	0.85
**Obstructive sleep apnea**	1.27 (0.79–2.08)	0.32	1.31 (0.79–2.18)	0.30
**CAD**	1.35 (0.92–1.97)	0.12	1.36 (0.90–2.05)	0.15
**Sodium**	0.98 (0.93–1.02)	0.30	0.97 (0.92–1.02)	0.29
**LVEF**	1.00 (0.99–1.01)	0.97	1.00 (0.99–1.02)	0.36

HR = hazard ratio, CAD = coronary artery disease, LVEF = left ventricular ejection fraction, PASP = pulmonary artery systolic pressure

**Fig 2:**
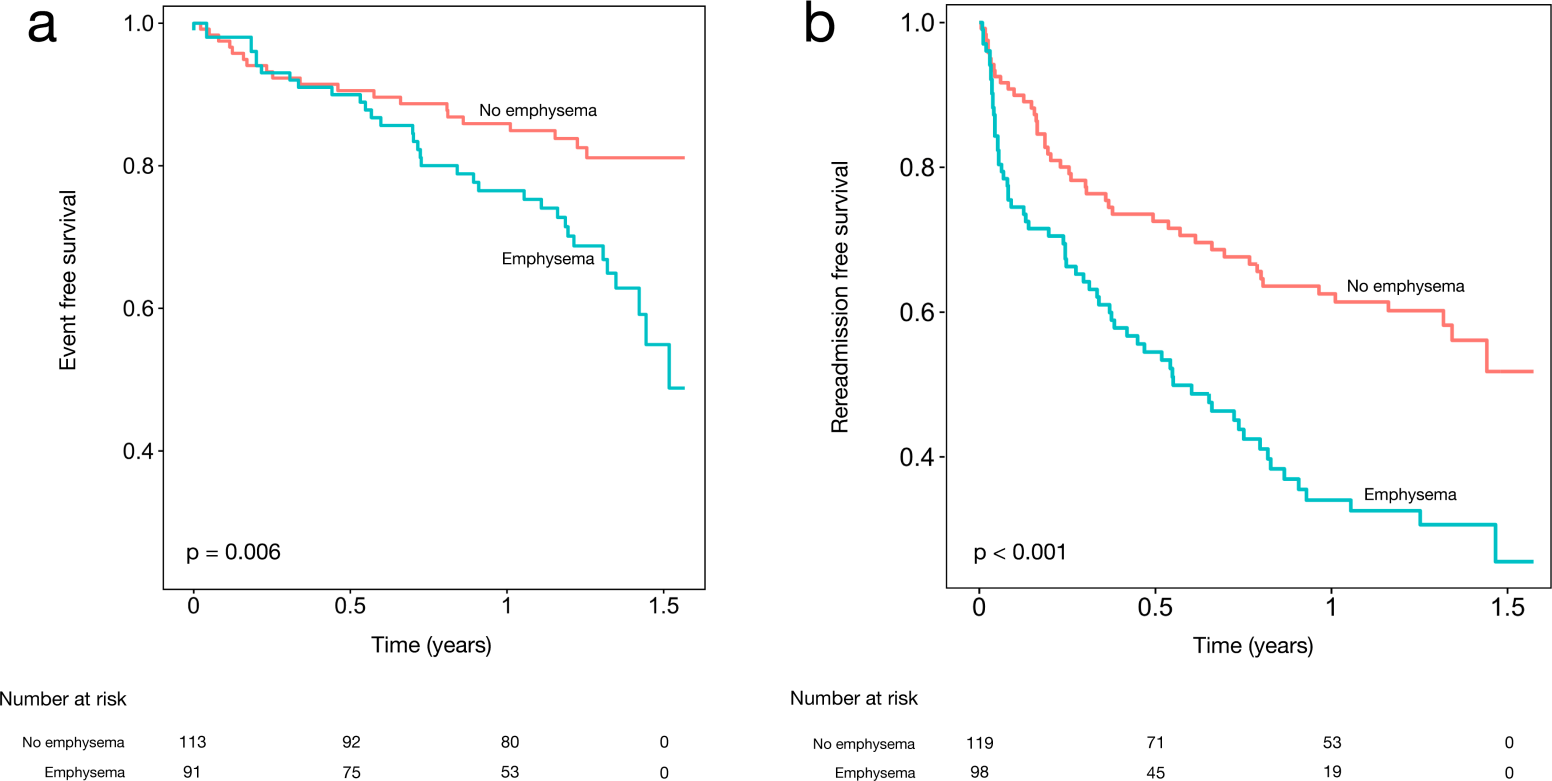
**Emphysema on CT is associated with increased mortality (a) and readmissions (b)**. Kaplan-Meier survival curves for mortality (a) and readmission free survival (b) showing that patients with emphysema on CT have decreased event free survival and decreased readmission free survival as compared to patients without emphysema on CT.

Emphysema on CT was associated with mortality in the univariate model (Table 7, HR 2.11, 95% CI 1.21–3.68, p < 0.001), along with age, sodium and PASP. In a multivariable model incorporating univariate predictors of mortality (i.e., age, sodium, and PASP), the presence of emphysema was found to be a trend toward increased mortality (Table 7, adjusted HR 1.70, 95% CI 0.86–3.34, p = 0.12). In a second multi-variable model, adjusting for factors known to be predictive of mortality, the presence of emphysema on CT was independently associated with mortality (Table 7, adjusted HR 2.10, 95% CI 1.16–3.76, p = 0.01). Kaplan-Meier curves generated for readmissions and event-free survival among patients with and without emphysema are presented (Fig 2).

**Table 7:** Cox proportional hazards models for time to mortality.

	Univariate analysis		Multivariate analysis			
			Model 1		Model 2	
	HR (95% CI)	p Value	HR (95% CI)	p Value	HR (95% CI)	p Value
**Emphysema**	2.11 (1.21–3.68)	0.008	1.70 (0.86–3.34)	0.12	2.10 (1.16–3.76)	0.01
**Age**	1.05 (1.02–1.08)	0.002	1.04 (1.00–1.08)	0.05	1.05 (1.02–1.08)	0.001
**Sex**	1.00 (0.56–1.77)	0.99	-	-	1.06 (0.56–2.00)	0.86
**Hypertension**	0.89 (0.49–1.61)	0.70	-	-	0.89 (0.46–1.73)	0.73
**Diabetes mellitus**	1.14 (0.66–1.94)	0.64	-	-	1.17 (0.67–2.06)	0.57
**Chronic kidney disease**	1.38 (0.78–2.43)	0.26	-	-	1.79 (0.97–3.28)	0.06
**Obstructive sleep apnea**	1.31 (0.67–2.53)	0.43	-	-	1.20 (0.60–2.40)	0.60
**CAD**	1.24 (0.72–2.14)	0.43	-	-	1.02 (0.57–1.83)	0.94
**Sodium**	0.93 (0.87–0.98)	0.01	0.87 (0.80–0.95)	0.002	0.92 (0.86–0.98)	0.01
**LVEF**	1.00 (0.98–1.01)	0.65	-	-	0.99 (0.98–1.01)	0.57
**PASP**	1.03 (1.01–1.05)	0.006	1.03 (1.00–1.05)	0.007	-	-

HR = hazard ratio, CAD = coronary artery disease, LVEF = left ventricular ejection fraction, PASP = pulmonary artery systolic pressure

## Discussion

In this study, we determined the associations between emphysema on chest CT and baseline variables among a consecutive group of smokers who were hospitalized with HF and subsequently tested the association between the presence of emphysema on chest CT and outcomes among these patients. The following were the principal findings: 1. Emphysema by CT is highly prevalent among prior or active smokers admitted with HF; 2. 40 (39%) prior or active smokers with HF who had emphysema on CT did not have a clinical diagnosis of COPD; 3. The presence of emphysema is associated with a markedly increased afterload; 4. The presence of emphysema among patients with HF provided prognostic information, with emphysema being associated with increased readmission for HF, a longer length of stay, and increased mortality.

These data on the high rate of adverse events among prior or active smokers with HF are complimentary and additive to prior data. For example, in the observational Worcester Heart Failure Study, containing 9,748 patients hospitalized with HF, patients with clinical COPD, the combination of emphysema and bronchitis, had a significantly higher mortality at 1 year [22]. In the Heart Failure and A Controlled Trial Investigating Outcomes of Exercise TraiNing (HF-ACTION) study, subjects with an LVEF of < 35% were randomized to usual care with or without aerobic exercise training; 249 patients had a clinical diagnosis of COPD. During follow-up, COPD was associated with a 1.5 fold increased risk of the combined end-point of CV mortality and HF hospitalization [41]. In our contemporary study where most individuals were on standard HF therapies, we found that the risk of readmission and mortality was high among this population. Specifically, over the duration of this study, approximately 50% of patients were readmitted, the 30-day readmission rate was 36 (16%) and 24% of patients died. These data suggest that additional awareness, biomarkers, and additional therapies may be of use to help both stratify and treat this vulnerable population.

A major diagnostic and therapeutic challenge in this group of patients with comorbid disease is teasing out whether their symptoms are due to pulmonary edema from heart failure or an acute exacerbation of obstructive airways disease. In our study, researchers blinded to all other variables used well established criteria to confirm the diagnosis of heart failure, including the use of echocardiographic features of left ventricular dysfunction. However, since these two diseases present with similar symptoms and often similar acute cardiopulmonary dysfunction, misclassification of the process considered to be the precipitant factor for hospitalization is always a possibility. Our current diagnostic tools including echocardiography [42,43], N-terminal pro-BNP [44] and pulmonary function testing [3] have variable accuracy in diagnosing exacerbations of HF and/or COPD in patients with comorbid illness. All of these tools require more robust studies to determine the diagnostic accuracy and utility in the complex HF patient population.

Computerized tomography is a sensitive and specific method for the diagnosis of emphysema; however, prior to this study, there was no prognostic information on the effect of emphysema by CT on outcomes specific to patients with HF. In the majority of studies, COPD is diagnosed using PFTs. However, the use of PFTs among patients with HF is limited. For example, in a study of 214 patients with valve disease and HF who never smoked, PFTs incorrectly diagnosed COPD in almost 45% of patients [45]. Additionally, in a study among non-smokers with HF, serial PFTs incorrectly diagnosed COPD in over 50% despite treatment and resolution of decomposition [4]. We found emphysema was present in almost 50% of smokers with HF and 39% without known COPD. We also found that the presence of emphysema by CT was associated with both HF readmission and length of stay for HF hospitalization. Based on our findings, the presence of emphysema by CT predicted adverse outcomes in complex HF.

The mechanism by which emphysema on CT is associated with increased morbidity and mortality among patients with HF is not completely understood. In broad populations without known HF, emphysema is associated with several pathophysiological changes that are likely detrimental among patients with HF. Specifically, emphysema is associated with an increased afterload, greater LV mass [14], higher pulmonary artery pressures and a lower cardiac output with a similar LVEF [15]. Relatively few studies have been designed to explore the possible pathophysiologic mechanisms underlying poor cardiovascular outcomes in this complex population living with COPD and HF. A study by Alter et al [46] found that in a cohort of patients with comorbid COPD and HF, increased LV wall stress by cardiac magnetic resonance imaging correlated with increasing severity of airflow obstruction as measured by reduced FEV1 and decreased FEV1/FVC ratio. This group and others postulate that the negative pleural pressure caused by inspiring against increased airways resistance is transmitted to the heart causing increased transmural pressure leading to increased wall stress. Furthermore, a subsequent study by Stone et al [47] showed that pharmacologic lung deflation via decreased airways resistance in patients with hyperinflated COPD improved left and right heart function in addition to pulmonary artery pulsatility. The decompression of the heart and associated pulmonary vasculature lead to normalization of pre-load and improvement in stroke volume. Inflammation may also contribute to poor cardiovascular outcomes in patients with comorbid disease. Specifically, systemic inflammation has been shown to be three-fold higher in COPD without HF [48] and greater than 2-fold higher among patients with HF alone when compared to healthy subjects [49]. Some hypothesize that low grade systemic inflammation in COPD accelerates progression of coronary atherosclerosis, ultimately leading to ischemic cardiomyopathy [50]. More recently, low level systemic inflammation and muscle disuse in the setting of worsening dyspnea has been linked to similar skeletal muscle atrophy in both HD and COPD, which can contribute to morbidity including increasing dypsnea and functional intolerance [51–53].

In this study, we also found that the LVEF was similar between groups with and without emphysema but did not find differences in left ventricular wall thickness or pulmonary artery pressures. However, we did find that LV afterload was markedly elevated among patients with emphysema and HF, which likely contributes to the complex physiologic changes that lead to poor outcomes in this population. There are no data testing the effect of emphysema by CT on LV afterload among patients with HF. Arterial function, as a measure of afterload, is increased in COPD and is associated with the severity of airflow limitation [54,55]. Our study findings add to that data and show an association between emphysema by CT and an increased cardiac afterload specifically among patients with HF. The mechanism by which afterload is increased in patients with emphysema and HF is unclear; however, data suggest that increased transmural pressure due to thoracic pressure swings, extrinsic compression in the setting of hyperinflation, and systemic inflammation may all play a role.

This study has to be interpreted within the study limitations. Specifically, PFTs were not available in all patients. The subset sample size was large enough to appropriately detect a difference in the FEV1/FVC ratio but may not have afforded the power to detect a difference in all pulmonary function measurements between those with and without emphysema on CT scan. Pulmonary function testing is generally considered the gold-standard for the diagnosis of emphysema; however, consistent data have acknowledged difficulty in interpretation of PFT’s among patients with HF. Additionally, as this was a retrospective study, we did no assay serum biomarkers such as NT-proBNP and BNP. These markers have been shown to be elevated in HF and to be strong predictors of adverse outcomes in HF. However, the data in COPD are conflicting; specifically, natriuretic peptide levels have been shown to be a predictor of adverse events in COPD without but not with HF [56–58].

In conclusion, among smokers hospitalized with HF, we found that emphysema on chest CT was highly prevalent and associated with increased afterload and adverse HF events. These data should serve as the basis for further study including considering whether therapy based on the presence of emphysema on CT can improve complex HF outcomes. Specifically, future studies should consider whether initiation of anti-muscarinics, beta-agonists or inhaled steroids based on the presence of emphysema on chest CT can improve complex HF outcomes.

## Supporting information

S1 Table Individual readmission and mortality data based on emphysema on CT and ejection fraction. (CSV)

## Abbreviations

ACEI: angiotensin-converting enzyme inhibitor
ARB: angiotensin II receptor blocker
BMI: body mass index
HF: heart failure
COPD: chronic obstructive pulmonary disease
LVEF: left ventricular ejection fraction
ECG: electrocardiogram
